# Rietveld refinement of Ba_5_(AsO_4_)_3_Cl from high-resolution synchrotron data

**DOI:** 10.1107/S1600536808026901

**Published:** 2008-08-23

**Authors:** Anthony M. T. Bell, C. Michael B. Henderson, Richard F. Wendlandt, Wendy J. Harrison

**Affiliations:** a Synchrotron Radiation Source, STFC Daresbury Laboratory, Daresbury, Warrington, Cheshire, WA4 4AD, England; b School of Earth, Atmospheric and Environmental Sciences, University of Manchester, Manchester, M13 9PL, England; c Department of Geology and Geological Engineering, Colorado School of Mines, Golden, CO 80401, USA

## Abstract

The apatite-type compound Ba_5_(AsO_4_)_3_Cl, penta­barium tris­[arsenate(V)] chloride, has been synthesized by ion exchange at high temperature from a synthetic sample of mimetite (Pb_5_(AsO_4_)_3_Cl) with BaCO_3_ as a by-product. The results of the Rietveld refinement, based on high resolution synchrotron X-ray powder diffraction data, show that the title compound crystallizes in the same structure as other halogenoapatites with general formula *A*_5_(*Y*O_4_)_3_ *X* (*A* = divalent cation, *Y* = penta­valent cation, *X* = Cl, Br) in space group *P*6_3_/*m*. The structure consists of isolated tetra­hedral AsO_4_^3−^ anions (*m* symmetry), separated by two crystallographically independent Ba^2+^ cations that are located on mirror planes and threefold rotation axes, respectively. The Cl^−^ anions are at the 2*b* sites (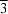 symmetry) and are located in the channels of the structure.

## Related literature

For crystal chemistry of apatites, see: Mercier *et al.* (2005 ▶); White & ZhiLi (2003 ▶); Wu *et al.* (2003 ▶). For powder diffraction data on Ba-containing As-apatites, see: Kreidler & Hummel (1970 ▶); Dunn & Rouse (1978 ▶). Atomic coordinates as starting parameters for the Rietveld (Rietveld, 1969 ▶) refinement of the present phases were taken from Chengjun *et al.* (2005 ▶); Dai *et al.* (1991 ▶); de Villiers *et al.* (1971 ▶). For related Ba—Cl-apatites, see: Đordevic *et al.* (2008 ▶); Hata *et al.* (1979 ▶); Reinen *et al.*(1986 ▶); Roh & Hong (2005 ▶); Schiff-Francois *et al.* (1979 ▶). For synthetic work, see: Baker (1966 ▶); Essington (1988 ▶); Harrison *et al.* (2002 ▶).

## Experimental

### 

#### Crystal data

As_3_Ba_5_ClO_12_
*M* *_r_* = 1138.85
Hexagonal, 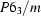

*a* = 10.5570 (1) Å
*c* = 7.73912 (8) Å
*V* = 746.98 (1) Å^3^
*Z* = 2
Synchrotron radiation
λ = 0.998043 Å
μ = 56.07 (1) mm^−1^
*T* = 298 K
Specimen shape: cylinder
40 × 0.7 × 0.7 mm
Specimen prepared at 100 kPa
Specimen prepared at 1258 K
Particle morphology: powder, white

#### Data collection

In-house design diffractometer
Specimen mounting: capillary
Specimen mounted in transmission mode
Scan method: step
Absorption correction: none
2θ_min_ = 2, 2θ_max_ = 70°
Increment in 2θ = 0.01°

#### Refinement

*R*_p_ = 0.059
*R*_wp_ = 0.082
*R*_exp_ = 0.067
*R*_B_ = 0.090
*S* = 1.23
Excluded region(s): 2-6 degrees 2θ.
Profile function: Fundamental Parameters
464 Bragg reflections
21 parameters
Preferred orientation correction: none

### 

Data collection: local software; cell refinement: *CELREF* (Laugier & Bochu, 2003 ▶); data reduction: local software; method used to solve structure: coordinates taken from a related compound; program(s) used to refine structure: *TOPAS* (Coelho, 2000 ▶); molecular graphics: *Balls and Sticks* (Kang & Ozawa, 2003 ▶); software used to prepare material for publication: *publCIF* (Westrip, 2008 ▶).

## Supplementary Material

Crystal structure: contains datablocks global, I. DOI: 10.1107/S1600536808026901/wm2188sup1.cif

Rietveld powder data: contains datablocks I. DOI: 10.1107/S1600536808026901/wm2188Isup2.rtv

Additional supplementary materials:  crystallographic information; 3D view; checkCIF report

## Figures and Tables

**Table:** 

Ba1—O1	2.67 (5)
Ba1—O2^i^	2.81 (4)
Ba1—O3^i^	3.12 (3)
Ba2—O2^ii^	2.59 (4)
Ba2—O3^iii^	2.62 (4)
Ba2—O3^iv^	3.05 (4)
Ba2—O1^v^	3.14 (4)
Ba2—Cl1^iv^	3.281 (5)
As1—O3	1.64 (2)
As1—O1	1.70 (8)
As1—O2	1.70 (4)

**Table:** 

O3—As1—O3^vi^	118 (2)
O3—As1—O1	108 (1)
O3—As1—O2	108 (2)
O1—As1—O2	106 (2)

Symmetry codes: (i) 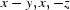; (ii) 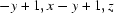; (iii) 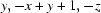; (iv) 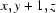; (v) 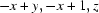; (vi) 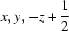.

**Fig. 1.**
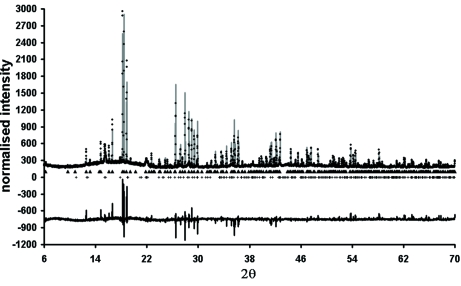
Rietveld difference plot for the multi-phase refinement of Ba5(AsO4)3Cl and BaCO3. The black dots, and grey and black lines show respectively the observed, calculated and difference plots. Calculated Bragg reflection positions are indicated by triangles for the Ba5(AsO4)3Cl phase and by crosses for the BaCO3 phase.

**Fig. 2.**
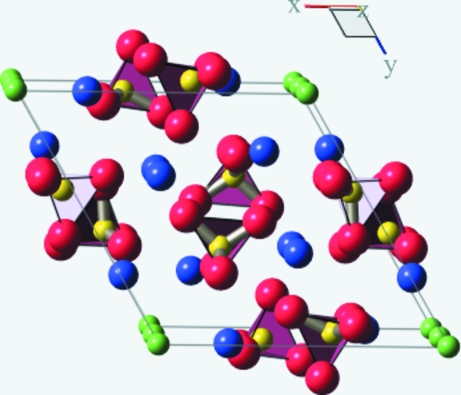
The crystal structure of Ba5(AsO4)3Cl. Pink tetrahedra show AsO4 units with As5+ cations as yellow spheres and O2- anions as red spheres. Large blue spheres represent Ba2+ cations and small green spheres Cl- anions.

## supplementary crystallographic information

### Comment

Apatites are minerals and synthetic compounds with general formula *A*_5_(*Y*O_4_)_3_*X*, containing tetrahedrally coordinated *Y*O_4_^3-^ anions (*Y* = pentavalent cation) and a monovalent anion *X* such as F^-^, Cl^-^ or OH^-^. The divalent cations frequently belong to the alkaline earth group, but other cations like Pb^2+^ are also known. For a review of the structures and crystal-chemistry of these materials, see Mercier *et al.* (2005) and White & Dong (2003). Apatites containing arsenic (As-apatites) are of interest as hosts for storage of arsenic removed from contaminated water (Harrison *et al.*, 2002). Powder diffraction data for the Ba containing As-apatites Ba_5_(AsO_4_)_3_Cl (Kreidler & Hummel, 1970) and for (Ba_2.25_Ca_1.65_Pb_1.16_Fe_0.06_Mg_0.06_)[(AsO_4_)_2.56_(PO_4_)_0.3_]Cl_1.09_ (mineral name morelandite; Dunn & Rouse, 1978) were indexed in space group *P*6_3_/*m*. Related crystal structures have also been reported for Ba_5_(AsO_4_)_2_SO_4_S (Schiff-Francois *et al.*, 1979) and (Sr_1.66_Ba_0.34_)(Ba_2.61_Sr_0.39_)(AsO_4_)_3_Cl (Dordevic *et al.*, 2008). The crystal structure of Ba_5_(AsO_4_)_3_Cl in space group *P*6_3_/*m* is reported in the present communication.

Table 1 shows refined interatomic distances and angles for the Ba_5_(AsO_4_)_3_Cl structure. The averaged Ba1—O and Ba2—O distances of respectively 2.87 Å and 2.84 Å are similar to those in other Ba and Cl containing apatites. In comparison, the average Ba1—O and Ba2—O distances are 2.84 Å and 2.78 Å for Ba_5_(VO_4_)_3_Cl (Roh & Hong, 2005), 2.83 Å and 2.79 Å for Ba_5_(PO_4_)_3_Cl (Hata *et al.*, 1979) and 2.83 Å and 2.76 Å for Ba_5_(MnO_4_)_3_Cl (Reinen *et al.*, 1986). The As—O distances are characteristic for tetrahedral AsO_4_ units. The O—As—O angles deviate significantly from the ideal tetrahedral angle of 109.5°, indicating a strong distortion.

The refined lattice parameters for Ba_5_(AsO_4_)_3_Cl are similar to the previously published parameters of a = 10.54 Å, c = 7.73 Å given by Kreidler & Hummel (1970). A study of 108 existing and predicted apatites with different compositions made use of elemental radii to calculate their lattice parameters (Wu *et al.*, 2003). Only 52 of these compositions had known lattice parameters. The predicted lattice parameters for Ba_5_(AsO_4_)_3_Cl were a = 10.3979 Å, c = 7.6105 Å. These predicted parameters are respectively 1.51% and 1.66% smaller than the measured lattice parameters, and only 2 of the 52 apatite compositions had bigger differences between observed and calculated lattice parameters.

Fig. 1 shows the Rietveld difference plot for the present refinement. The crystal structure of Ba_5_(AsO_4_)_3_Cl, showing the isolated tetrahedral AsO_4_^3-^ anions separated by Ba^2+^ cations and Cl^-^ anions, is displayed in Fig. 2.

### Experimental

This work was part of an attempt to synthesize analogues of Pb_5_(AsO_4_)_3_Cl (mimetite) with Pb^2+^ substituted by alkaline earth cations. All starting materials were well crystallized solids. Pb_5_(AsO_4_)_3_Cl was precipitated by titration of 0.1*M* Na_2_HAsO_4_ into a well stirred, saturated PbCl_2_ solution at room temperature (procedure modified from methods of Baker (1966) and Essington (1988)). The molar ratio of Pb:As was slightly greater than 5:3, allowing for excess PbCl_2_ during the precipitation. A very fine-grained pure solid formed immediately, which was then separated, washed, and dried. Typically, five de-ionized water washes were needed to reduced the conductivity of the wash water to < 50 µS^.^cm^-1^. Ba_5_(AsO_4_)_3_Cl was successfully synthesized by ion exchange of Pb_5_(AsO_4_)_3_Cl with molten BaCl_2_ at 1258 K (modified from the method given by Kreidler & Hummel (1970)). Two fusions were required to completely eliminate formation of Pb containing solid solutions and to yield the Pb free title compound. Excess metal in the form of BaCl_2_ was removed from the solids by repeated washing with de-ionized water followed by centrifugation and filtration to separate the solid from the solution.

### Refinement

The powdered sample was loaded into a 0.7 mm diameter borosilicate capillary, prior to high-resolution synchrotron X-ray powder diffraction data collection using station 9.1 of the Daresbury Synchrotron Radiation Source. The beam on the sample was 13 mm wide and 1.2 mm high. 9 powder datasets were collected, all were with a step with of 0.01°/2θ and a counting time of 2 s per point. Three of these datasets were collected between 5–70°/2θ, two between 30–70°/2θ, two between 40–70°/2θ, one between 31.73–70°/2θ and one between 2–13.2°/2θ. All of these data were summed and normalized to account for decay of the synchrotron beam with time. The main Bragg reflections of the powder diffraction pattern could be indexed in space group *P*6_3_/*m* with similar lattice parameters to those of the published powder diffraction data (Kreidler & Hummel, 1970). Some broad and weak Bragg reflections were matched by the pattern of BaCO_3_ in space group *Pmcn*. The synchrotron X-ray wavelength was calibrated as 0.998043Å with an external *NIST* 640*c* silicon standard reference material.

Initial lattice parameters for the two phases were refined using *CELREF* (Laugier & Bochu, 2003). The *P*6_3_/*m* crystal structure of Ba_5_(PO_4_)_3_(OH) (Chengjun *et al.*, 2005) was used as a starting model for the Rietveld (Rietveld, 1969) refinement of the structure of Ba_5_(AsO_4_)_3_Cl. The crystal structure of witherite (de Villiers *et al.*,  1971) was used as a starting model for refinement of the structure of BaCO_3_. Isotropic atomic displacement parameters were used for both phases. For the Ba_5_(AsO_4_)_3_Cl phase the As—O distances in the AsO_4_ tetrahedral units were constrained to those for mimetite (Dai *et al.*, 1991). For the BaCO_3_ phase the C—O distances of the trigonal carbonate anion were constrained to those in witherite, and the U_iso_ factors for all atoms in the carbonate anion were constrained to be the same. As the Ba_5_(AsO_4_)_3_Cl phase was prepared by ion-exchange of Pb_5_(AsO_4_)_3_Cl, Rietveld refinements were done with the metal sites partially occupied by both Pb and Ba. However, this resulted in the refined Pb occupancies falling to zero. Therefore the occupancies of the metal sites were fixed as fully occupied by Ba and no Pb was included for the final refinement of the Ba_5_(AsO_4_)_3_Cl phase. Proportions of the two phases were refined as 64.7 (9) wt.% Ba_5_(AsO_4_)_3_Cl and 35.3 (9) wt.% BaCO_3_.

### Crystal data

**Table:**

As_3_Ba_5_Cl_1_O_12_	*Z* = 2
*M**_r_* = 1138.85	*D*_x_ = 5.063 (1) Mg m^−^^3^
Hexagonal, *P*6_3_/*m*	Synchrotron radiation λ = 0.998043 Å
*a* = 10.5570 (1) Å	µ = 56.07 (1) mm^−^^1^
*b* = 10.5570 (1) Å	*T* = 298 K
*c* = 7.73912 (8) Å	Specimen shape: cylinder
α = 90º	40 × 0.7 × 0.7 mm
β = 90º	Specimen prepared at 100 kPa
γ = 120º	Specimen prepared at 1258 K
*V* = 746.98 (1) Å^3^	Particle morphology: powder, white

### Data collection

**Table:**

In-house design diffractometer	*T* = 298 K
Monochromator: Si(111) channel-cut crystal	2θ_min_ = 2, 2θ_max_ = 70º
Specimen mounting: capillary	Increment in 2θ = 0.01º
Specimen mounted in transmission mode	Increment in 2θ = 0.01º
Scan method: step	

### Refinement

**Table:**

*R*_p_ = 0.059	Profile function: Fundamental Parameters
*R*_wp_ = 0.082	21 parameters
*R*_exp_ = 0.067	3 constraints
*R*_B_ = 0.090	?
*S* = 1.23	(Δ/σ)_max_ = 0.001
Wavelength of incident radiation: 0.998043 Å	Preferred orientation correction: None
Excluded region(s): 2-6 degrees 2θ.	

### Fractional atomic coordinates and isotropic or equivalent isotropic displacement parameters (Å^2^)

**Table:**

	*x*	*y*	*z*	*U*_iso_*/*U*_eq_	
Ba1	0.3333	0.6667	0.0061 (9)	0.059 (1)	
Ba2	0.2445 (4)	0.9874 (6)	0.2500	0.065 (1)	
As1	0.4047 (7)	0.3716 (7)	0.2500	0.059 (2)	
O1	0.347 (7)	0.495 (6)	0.2500	0.13 (2)	
O2	0.591 (4)	0.473 (4)	0.2500	0.08 (1)	
O3	0.354 (2)	0.280 (3)	0.068 (3)	0.065 (8)	
Cl1	0.0000	0.0000	0.0000	0.070 (6)	

### Geometric parameters (Å, °)

**Table:**

Ba1—O1^i^	2.67 (5)	Ba2—O3^vi^	2.62 (4)
Ba1—O1^ii^	2.67 (5)	Ba2—O3^vii^	3.05 (4)
Ba1—O1	2.67 (5)	Ba2—O3^viii^	3.05 (4)
Ba1—O2^iii^	2.81 (4)	Ba2—O1^ii^	3.14 (4)
Ba1—O2^iv^	2.81 (4)	Ba2—Cl1^viii^	3.281 (5)
Ba1—O2^v^	2.81 (4)	Ba2—Cl1^ix^	3.281 (5)
Ba1—O3^iv^	3.12 (3)	As1—O3	1.64 (2)
Ba1—O3^iii^	3.12 (3)	As1—O3^x^	1.64 (2)
Ba1—O3^v^	3.12 (3)	As1—O1	1.70 (8)
Ba2—O2^i^	2.59 (4)	As1—O2	1.70 (4)
Ba2—O3^iv^	2.62 (4)		
			
O3—As1—O3^x^	118 (2)	O3—As1—O2	108 (2)
O3—As1—O1	108 (1)	O3^x^—As1—O2	108 (2)
O3^x^—As1—O1	108 (1)	O1—As1—O2	106 (2)

Symmetry codes: (i) −*y*+1, *x*−*y*+1, *z*; (ii) −*x*+*y*, −*x*+1, *z*; (iii) *x*−*y*, *x*, −*z*; (iv) *y*, −*x*+*y*+1, −*z*; (v) −*x*+1, −*y*+1, −*z*; (vi) *y*, −*x*+*y*+1, *z*+1/2; (vii) *x*, *y*+1, −*z*+1/2; (viii) *x*, *y*+1, *z*; (ix) −*x*, −*y*+1, *z*+1/2; (x) *x*, *y*, −*z*+1/2.
